# Quality, readability, and patient safety of ChatGPT-generated responses to fall-related questions in older adults: a multidisciplinary evaluation

**DOI:** 10.1590/1806-9282.20260388

**Published:** 2026-08-24

**Authors:** Merve Arı, Nursen İlçin, Hatice Yağcıoğlu, İlyas Akkar

**Affiliations:** 1 KTO Karatay University, Vocational School of Health Services, Department of Medical Services and Techniques, Medical Imaging Techniques Program – Konya, Türkiye.; 2 Dokuz Eylul University, Health Sciences Institute – İzmir, Türkiye.; 3 Dokuz Eylul University, Faculty of Physical Therapy and Rehabilitation, Department of Physiotherapy and Rehabilitation, Division of Geriatric Physiotherapy – İzmir, Türkiye.; 4 Pamukkale University, Graduate School of Health Sciences, Department of Anatomy – Denizli, Türkiye.; 5 University of Health Sciences, Konya City Hospital, Department of Internal Medicine, Division of Geriatrics – Konya, Türkiye.

**Keywords:** Aged, Accidental falls, Artificial intelligence, Large language models, Patient safety, Health literacy

## Abstract

**OBJECTIVE:**

Older adults increasingly use artificial intelligence-based tools to obtain health information. Although artificial intelligence chatbots such as ChatGPT may enhance access, the quality, readability, and patient safety of fall-prevention information remain uncertain. This study aimed to evaluate the quality, readability, and patient safety implications of ChatGPT-generated responses to common questions about fall risk and home safety in older adults.

**METHODS:**

Ten frequently asked fall-related questions were submitted to ChatGPT (version 5.2). Responses were independently assessed by a multidisciplinary panel including physiotherapists, a geriatrician, a physical medicine and rehabilitation physician, an occupational therapist, and an orthopedic specialist. Quality was evaluated using the Mika classification. Readability was measured with the Flesch-Kincaid Grade Level. Interrater reliability was analyzed using a two-way random-effects intraclass correlation coefficient model with absolute agreement (intraclass correlation coefficient [2,k]).

**RESULTS:**

Three responses were rated as "excellent," while seven responses were rated as "satisfactory requiring minimal clarification." No response received a rating corresponding to "moderately satisfactory" or "unsatisfactory." The mean Flesch-Kincaid Grade Level was 8.4 (range 4.3–11.9). Five responses exceeded the readability levels commonly recommended for patient education materials. Interrater reliability demonstrated fair agreement (intraclass correlation coefficient [2,k]=0.72; 95%CI 0.64–0.80).

**CONCLUSION:**

While ChatGPT provided generally acceptable clinical information, variability in readability and expert ratings raises patient safety concerns. AI-generated health content should be reviewed and tailored to older adults’ health literacy needs before clinical use.

## INTRODUCTION

Falls are one of the leading causes of injury, functional impairment, and mortality in older adults and represent a serious public health problem on a global scale^1^. Approximately one-third of individuals aged 65 years and older fall at least once a year, resulting in hip fractures, traumatic injuries, loss of independence, and the need for long-term care^2^. Healthcare expenditures related to falls are increasing and place a significant economic burden on healthcare systems^3^.

The etiology of falls is multifactorial. Muscle weakness, balance disorders, vision and hearing loss, cognitive decline, polypharmacy, and chronic diseases are key individual risk factors^4^. However, the majority of falls occur in the home environment; inadequate lighting, slippery floors, inappropriate bathroom and toilet arrangements, and the lack of grab bars are among the significant environmental risks^5^. Therefore, home safety measures are at the center of evidence-based fall prevention programs^6^.

The use of digital tools to access health information has increased significantly in recent years^7^. Artificial intelligence (AI)-based chatbots can generate quick responses to users’ medical questions and are seen as a potential patient education tool^8^. Studies examining ChatGPT's performance in the health field have reported that the model can provide understandable and consistent answers on many topics, but can vary in terms of clinical appropriateness and reliability^9^.

A recent study in the field of physical therapy and rehabilitation evaluated the accuracy of ChatGPT's responses to clinical questions and noted that the model generally performed acceptably, but had limitations in terms of source citation and level of evidence^10^. Similarly, another study examining the reliability of AI responses emphasized that while ChatGPT's answers to patient questions were mostly useful, they could not replace clinical decisions^11^. Studies addressing ChatGPT's potential in medical education and clinical decision support processes also stated that the model could serve as a supportive tool, but that expert supervision was mandatory^12^. Evaluations conducted in the field of the musculoskeletal system revealed that ChatGPT mostly provided satisfactory responses to orthopedic and rehabilitation-related questions^13^. In a study based on frequently asked questions about postural disorders, most of the responses were found to be adequate by experts, and the readability level was reported to be acceptable^14^. While this study demonstrates that AI-based tools can be used as a source of information in the field of rehabilitation, it emphasizes that the responses require scientific validation.

However, the quality, readability, and patient safety implications of ChatGPT responses on critical topics directly related to injury, such as fall risk and home safety in older adults, have not been sufficiently investigated. Incomplete or inaccurate information provided in this area could lead to irreversible clinical outcomes.

From a patient safety perspective, the use of AI-generated health information introduces several potential risks, particularly in the context of fall prevention in older adults. These risks may include incomplete or incorrect exercise recommendations, failure to recognize high-risk clinical conditions, and inability to identify situations requiring urgent medical evaluation, such as occult fractures following a fall. A major concern in this context is the phenomenon of "hallucination," defined as the generation of fluent but factually incorrect or unsupported information by large language models^15^.

Hallucinations have been identified as a structural limitation of large language models and represent a critical threat to reliability in healthcare applications. In medical contexts, such outputs may lead to inappropriate clinical reasoning, omission of essential safety information, or delayed diagnosis and treatment. Furthermore, AI-generated responses may lack adequate risk stratification and fail to appropriately guide users toward professional care, thereby increasing the likelihood of unsafe self-management. Given the high vulnerability of older adults to fall-related injuries, even minor inaccuracies in AI-generated recommendations may result in significant adverse clinical outcomes^16^.

In addition, health literacy plays a critical role in the safe use of digital health information among older adults. Age-related cognitive decline, reduced processing speed, and limited digital literacy may impair the ability to interpret and critically evaluate health-related content^17,18^. Previous studies have demonstrated that low health literacy is associated with poorer health outcomes, reduced adherence to preventive strategies, and increased risk of misinterpretation of medical information^17,19^. In the context of fall prevention, inadequate comprehension may result in improper execution of exercises, incorrect use of assistive devices, or failure to implement appropriate home safety measures.

The aim of this study is to evaluate the quality, readability, and patient safety implications of ChatGPT-generated responses to frequently asked questions about fall risk and home safety measures in older adults. It was hypothesized that although ChatGPT would provide generally acceptable clinical information, variability in response quality and readability could pose potential patient safety risks, particularly for older adults with limited health literacy.

## METHODS

### Research design

This study was designed as a descriptive study to evaluate the quality and readability of responses provided by ChatGPT regarding fall risk and home safety measures in older adults. Access to accurate and evidence-based information is critical for older adults in preventing falls.

### Question collection process

The following prompt was entered into the ChatGPT application: "Can you list the 50 most frequently asked questions about fall risk and home safety measures among older adults?" The generated question pool was reviewed in detail by two physical therapist researchers. A total of 10 questions were selected based on criteria of clinical relevance, specificity to the elderly population, and relevance to home safety (Table 1).

**Table 1:** Questions used in the study.

No	Question
1	Why do I fall more easily as I get older?
2	Does having fallen before increase my risk of falling again?
3	Should I wear slippers or shoes inside the house?
4	Should I use a cane or a walker inside the house?
5	How can I get up from bed or a chair more safely?
6	Am I using my cane at the correct height?
7	What exercises can I do to prevent falls?
8	Who can assess my home for fall risks?
9	If I don't have pain after falling and getting up, is that okay?
10	Where can I get professional support to prevent falls?

Note: The questions were selected by two physical therapists based on clinical appropriateness criteria.

The selection of 10 questions was based on methodological considerations to balance the depth of expert evaluation with feasibility. Given that each response was assessed by six independent experts, increasing the number of questions would have substantially increased evaluator burden and potentially reduced scoring consistency. Similar studies evaluating AI-generated medical content have used comparable sample sizes to allow detailed qualitative and quantitative assessment while maintaining reliability. This approach ensured reproducibility and reduced subjectivity in the question selection process.

To ensure methodological transparency, clinical relevance was operationalized using a structured scoring framework developed by the research team. Each question was independently rated by two physiotherapists across four domains:

1. Direct association with fall risk,
2. Relevance to home safety and daily living,
3. Frequency of occurrence in clinical practice, and
4. Need for professional guidance or clinical interpretation.

Each domain was scored on a 3-point scale (1=low relevance, 2=moderate relevance, and 3=high relevance), yielding a total score ranging from 4 to 12. Questions with the highest total scores were selected for inclusion. In cases of disagreement, consensus was achieved through discussion (Table 2).

**Table 2:** Distribution of fall-prevention subtopics represented by the selected questions.

Fall-prevention domain	Question numbers	Number
Fall-risk awareness	1, 2	2
Home safety and daily living	3, 5	2
Assistive device use	4, 6	2
Exercise and physical function	7	1
Professional assessment/support	8, 10	2
Post-fall management	9	1

### ChatGPT usage

The questions were posed using ChatGPT version 5.2. To prevent the model from being influenced by previous interactions, the browser history was cleared, a new ChatGPT account was created, the 10 questions were asked one by one in sequence, the initial responses were recorded, and no additional questions or prompts were made. The responses generated by ChatGPT did not include formal scientific citations or reference lists, and citation accuracy was therefore not evaluated as part of the study.

### Evaluation

The quality of the responses was evaluated by six independent experts: two physical therapists, one occupational therapist, one geriatric specialist, one orthopedic specialist, and one Physical Medicine and Rehabilitation physician.

The expert evaluators were not blinded to the source of the responses and were aware that the content was generated by an AI system. This design choice was intentional, as the study aimed to reflect real-world clinical appraisal of AI-generated patient information.

The four-level evaluation system proposed by Mika et al. was selected due to its clinical interpretability and prior use in assessing AI-generated responses to patient questions. Although originally applied in the context of total hip arthroplasty, the scale is not procedure-specific and is designed to evaluate the clinical adequacy, completeness, and need for clarification in patient-oriented information. Previous studies have adopted this framework in similar contexts of AI-based patient education, supporting its applicability. Therefore, no adaptation was required for fall-risk-related inquiries. The scale allows classification of responses based on their potential clinical usability, which aligns with the patient safety focus of the present study.

The four-level system proposed by Mika et al.^20^ was used in the evaluation:

1. Unsatisfactory response – the response contained substantial omissions, inaccuracies, or overly generalized statements that could potentially lead to misunderstanding or unsafe interpretation.
2. Moderately satisfactory response – the response contained appropriate information; however, further explanation was needed to ensure adequate understanding.
3. Satisfactory response requiring minimal clarification – the response was generally accurate but required limited supplementary information to fully address the question.
4. Excellent response – the response was considered clinically appropriate, comprehensive, and sufficiently clear without requiring additional explanation.

### Readability analysis

Each response was entered into WordCalc software, and the Flesch-Kincaid Grade Level (FKGL) value was calculated. This allowed for the assessment of the texts’ suitability for the health literacy level of older adults. The FKGL estimates the US school grade level required to comprehend a given text. Higher scores indicate greater reading difficulty.

### Interpretation of scores

- ≤5.0 → Very easy to read (elementary school level)
- 6.0–8.0 → Easy to read; consistent with recommended readability levels for patient education materials according to NIH and AMA guidelines^21,22^.
- 9.0–12.0 → Moderately difficult; high school reading level
- >12.0 → Difficult to read; college-level text

### Statistical analysis

Analyses were performed using the SPSS Statistics for Windows, Version 29.0 (IBM Corp., Armonk, NY, USA) program. Quality scores were given as median (min–max). The intraclass correlation coefficient (ICC) was used for inter-rater agreement^16^ (ICC interpretation ranges: <0.50 poor, 0.50–0.75 fair, 0.75–0.90 good, and 0.90 excellent).

## RESULTS

The study examined the readability of ChatGPT-generated responses to 10 frequently asked questions regarding fall risk and home safety measures in older adults. According to the Flesch-Kincaid analysis, five responses (50%) fell within the recommended sixth–eighth grade readability range, while the remaining five responses (50%) exceeded this range (Table 3).

**Table 3:** Readability and quality of ChatGPT responses.

Question number	FKGL	Median quality score	Min–max
1	6.3	3	2–4
2	6.8	4	3–4
3	5.9	3	2–4
4	4.3	3	3–4
5	7.4	3	3–4
6	5.4	3	2–4
7	6.2	3	2–3
8	9.7	4	3–4
9	6.6	4	3–4
10	11.9	3	2–4

FKGL: Flesch-Kincaid Grade Level. Overall interrater reliability demonstrated fair agreement among evaluators (ICC [2,k]=0.72; 95%CI 0.64–0.80).

The study was conducted using ChatGPT 5.2 in January 2026. Based on expert evaluation using the Mika classification, three responses were rated as "Excellent," while seven responses were rated as "Satisfactory, requiring minimal clarification." No response received a median rating corresponding to "Moderately satisfactory" or "Unsatisfactory."

The mean FKGL of the responses was 8.4, indicating moderate linguistic complexity overall (Table 3). Readability scores ranged from 4.3 to 11.9, with higher readability levels observed in responses involving technical explanations or professional support services.

Question-level analysis demonstrated variability in expert ratings across domains. Responses related to general fall mechanisms, previous fall history, and post-fall management were more frequently evaluated as excellent, whereas responses involving assistive device use and access to professional services showed greater variation in scoring.

Interrater reliability analysis demonstrated fair agreement among the evaluators, with an ICC (2,k) value of 0.72 (95%CI 0.64–0.80), supporting consistency in response quality assessment across different professional backgrounds.

## DISCUSSION

This study evaluated the quality, readability, and patient safety implications of ChatGPT-generated responses to fall-related questions in older adults. The findings indicate that while most responses were rated as satisfactory or excellent by a multidisciplinary expert panel, important concerns remain regarding readability consistency and potential patient safety risks.

Overall, expert evaluations demonstrated that ChatGPT was able to generate responses of generally satisfactory quality, with some responses rated as excellent. Notably, no response received a median rating corresponding to "moderately satisfactory" or "unsatisfactory," suggesting that the content produced by the model aligns broadly with current clinical understanding of fall risk and prevention in older adults. These findings suggest that AI-based tools may serve as supplementary sources of health information when used with appropriate professional oversight.

Nevertheless, clinically acceptable responses do not necessarily eliminate patient safety concerns. In several responses, the information provided was generally correct but lacked sufficient contextualization or risk stratification. For example, responses related to assistive device use, such as cane height adjustment or walker use, did not consistently emphasize the importance of individualized clinical assessment. Similarly, exercise-related recommendations occasionally lacked clear guidance regarding supervision, balance limitations, or contraindications in frail older adults. In addition, responses addressing post-fall situations could potentially be misinterpreted if users assume that the absence of pain excludes serious injury, despite the possibility of occult fractures or delayed complications in older individuals. Although these limitations were not severe enough to classify responses as "unsatisfactory," they represent subtle but clinically meaningful patient safety risks, particularly for users with low health literacy or limited clinical judgment.

However, the variability observed in expert ratings across certain questions highlights a critical issue. Differences in scoring likely reflect the multidisciplinary nature of the panel, as clinicians from different professional backgrounds may prioritize distinct aspects of fall prevention, such as functional capacity, environmental safety, or medical risk factors. Importantly, this variability does not undermine the reliability of the findings, as interrater agreement remained within an acceptable range, but rather underscores the complexity of fall prevention as a multifactorial clinical problem.

Readability analysis revealed clinically important concerns related to patient safety. Although the mean FKGL of 8.4 may be considered moderately complex in general populations, it exceeds the readability levels commonly recommended by NIH and AMA guidelines for patient education materials. These organizations generally recommend that health-related materials be written at approximately a sixth-grade reading level to maximize comprehension and accessibility.

In older adults, elevated readability levels may represent a substantial barrier to safe interpretation and application of health information due to age-related cognitive decline, reduced processing speed, and limited health literacy. Complex sentence structures, medical terminology, or insufficiently simplified instructions may increase the risk of misunderstanding, particularly in areas requiring practical decision-making, such as exercise implementation, assistive device use, or post-fall management. As a result, older individuals may incorrectly apply recommendations, delay seeking medical attention, or develop false reassurance regarding potentially serious conditions. Therefore, even clinically appropriate AI-generated information may pose indirect patient safety risks if readability is not adequately adapted to the target population.

Taken together, these findings suggest that while ChatGPT-generated responses may provide clinically relevant information, they should not be used as stand-alone patient education materials for fall prevention. Instead, AI-generated information may serve as an initial informational resource that requires professional review, simplification, and contextualization to ensure safe and effective use in older adults. Future research should explore whether targeted prompting strategies or health-literacy-optimized AI outputs can improve the safety and usability of such tools in geriatric care.

### Strengths and limitations

#### Strengths

A key strength of this study is its multidisciplinary evaluation framework, incorporating perspectives from physiotherapy, geriatrics, physical medicine and rehabilitation, occupational therapy, and orthopedic surgery. This approach reflects real-world clinical practice and enhances the relevance of the findings to geriatric care. In addition, the use of both quality assessment (Mika classification) and readability analysis provides a comprehensive evaluation of AI-generated health information beyond readability alone. The assessment of interrater reliability further strengthens the methodological rigor of the study.

#### Limitations

Several limitations should be acknowledged. First, the study evaluated a limited number of questions, which may not capture the full range of fall-related information sought by older adults. Second, responses were generated using a single version of ChatGPT at a specific time point; therefore, findings may not be generalizable to other AI models or future versions. Third, patient comprehension was inferred from readability formulas rather than direct testing with older adults.

Although the FKGL provides an objective estimate of textual complexity, it does not fully reflect actual understanding, interpretation, or practical application of health information in real-world settings. Factors such as cognitive decline, sensory impairment, educational background, and digital literacy may substantially influence how older adults interpret AI-generated recommendations. Therefore, the real-world clinical usability and comprehension of the responses cannot be fully established without direct user-based evaluation.

One important limitation of this study is that the expert evaluators were not blinded to the source of the responses. Awareness that the content was generated by an AI system may have introduced evaluation bias, potentially influencing scoring behavior either positively or negatively. Experts may have been more critical due to known limitations of AI systems or, conversely, more lenient due to expectations of technological performance. Therefore, the findings should be interpreted with caution, as evaluator perceptions may have affected the assessment of response quality. Future studies using blinded evaluation designs are warranted to better isolate the intrinsic quality of AI-generated responses.

Another limitation of this study relates to the use of the four-level evaluation system proposed by Mika et al. Although this scale has been applied in previous studies assessing AI-generated responses to patient questions, it was originally developed in a different clinical context and has not been specifically validated for fall-risk-related inquiries. While the scale provides a practical and clinically interpretable framework for evaluating response adequacy, it may not fully capture all dimensions relevant to fall prevention, such as risk stratification, contextual appropriateness, or patient-specific safety considerations. Future research may benefit from developing or adapting evaluation tools specifically tailored to fall-related patient education and safety outcomes.

Another limitation of this study is that the accuracy and authenticity of scientific citations were not evaluated. The ChatGPT-generated responses analyzed in this study were primarily patient-oriented and did not contain formal references or source citations. Although previous literature has highlighted concerns regarding AI hallucinations and fabricated references, the present study focused specifically on the clinical quality, readability, and patient safety implications of the generated content rather than citation validity.

## CONCLUSION

ChatGPT-generated responses to fall-related questions in older adults demonstrated generally acceptable clinical quality; however, variability in readability and expert evaluations raised important patient safety considerations. While AI-based chatbots may serve as supportive sources of health information, they should not replace professional guidance in fall prevention. Ensuring that AI-generated health content is adapted to the health literacy needs of older adults and reviewed by healthcare professionals is essential for its safe and effective use in geriatric care. Future studies should incorporate direct user testing with older adults to evaluate real-world comprehension, usability, and the safe implementation of AI-generated fall-prevention recommendations in clinical and home environments.

## Data Availability

The datasets generated and/or analyzed during the current study are available from the corresponding author upon reasonable request.
